# Obstructive sleep apnea in patients with interstitial lung disease: Prevalence and predictive factors

**DOI:** 10.1371/journal.pone.0239963

**Published:** 2020-10-05

**Authors:** Jae Ha Lee, Chan Sun Park, Jin Woo Song

**Affiliations:** 1 Division of Pulmonology, Department of Internal Medicine, Inje University Haeundae Paik Hospital, Inje University College of Medicine, Busan, Republic of Korea; 2 Division of Allergy, Department of Internal Medicine, Inje University Haeundae Paik Hospital, Inje University College of Medicine, Busan, Republic of Korea; 3 Department of Pulmonary and Critical Care Medicine, Asan Medical Center, University of Ulsan College of Medicine, Seoul, Republic of Korea; BronxCare Health System, Affiliated with Icahn School of Medicine at Mount Sinai, NY, USA, UNITED STATES

## Abstract

Interstitial lung diseases (ILDs) are chronic, progressive, parenchymal lung diseases with high morbidity and mortality. In recent studies, the prevalence of obstructive sleep apnea (OSA) in patients with ILD has been reported to be high. However, the prevalence and predictive factors of OSA in Korean ILD patients are not well defined. Therefore, the aim of this study was to evaluate the prevalence and predictive factors of OSA in Korean patients with ILD. Clinical data from 86 patients with ILD enrolled from December 2017 to April 2019 at Haeundae-Paik Hospital, Busan, South Korea, were retrospectively analyzed. OSA was monitored with a level 4 portable device and defined as an apnea-hypopnea index of more than 5 per hour of sleep. The median follow-up period was 7 months. The mean age was 69.8 years, and 64% of participants were men. Among the ILDs, idiopathic pulmonary fibrosis (IPF) was the most common (66.3%), followed by connective tissue disease–associated ILD (16.3%) and cryptogenic organizing pneumonia (5.8%). Forty-six ILD patients (53.5%) were diagnosed with OSA, and IPF patients had OSA more frequently (64.9% vs. 31.0%, p = 0.003) than those with other ILDs. Older age (odds ratio [OR], 1.11, 95% CI 1.04–1.19, p = 0.002), higher body weight (OR 1.05, 95% CI 1.01–1.10, p = 0.012), and diabetes mellitus (OR 4.03, 95% CI 1.26–12.91, p = 0.019) were independent risk factors for OSA in the multivariable logistic regression analysis. In the multivariable Cox analysis, an IPF diagnosis was a significant risk factor for one-year mortality (hazard ratio [HR] 7.92, 95% CI: 1.01–61.83, p = 0.048) in ILD patients; however, OSA was not. In conclusion, half of Korean patients with ILD had OSA. Older age, higher body weight, and diabetes mellitus were risk factors for OSA in patients with ILD.

## Introduction

Interstitial lung diseases (ILD) are a heterogeneous group of diffuse parenchymal lung disorders with highly variable clinical courses and outcomes [1]. Among the ILDs, idiopathic pulmonary fibrosis (IPF) is the most common type of idiopathic interstitial pneumonia and has a median survival time of 3–4 years [2, 3]. ILD patients often have comorbidities that significantly affect their clinical outcomes. Several comorbidities, including gastroesophageal reflux disease, pulmonary hypertension, depression, and obstructive sleep apnea (OSA), are known to be frequently associated with ILDs [4]. Especially among IPF patients, international guidelines from the American Thoracic Society, European Respiratory Society, Japanese Respiratory Society, and Latin American Thoracic Association have acknowledged the importance of comorbidities [5].

Among those comorbidities, an increasing number of studies is describing the importance of OSA in patients with ILD. The prevalence of OSA in ILD has been reported to range from 17–88%, compared with 2–4% in healthy adults [6–8]. OSA is characterized by repetitive episodes of upper airway collapse leading to apnea, hypopnea, intrathoracic pressure swings, intermittent hypoxia, and arousal from sleep due to consecutive activation of the sympathetic nervous system [9]. Recent studies have demonstrated that OSA in ILD patients is related to excessive daytime sleepiness, poor quality of life, morbidity, and premature mortality [10, 11]. In a previous study including middle-aged Koreans (40–69 years old), the prevalence of OSA was reported 27.1% in men and 16.8% in women [12]. However, the prevalence and predictive factors for OSA in Korean patients with ILD are not well defined. Therefore, our aim in this study was to evaluate the prevalence and predictive factors for OSA in Korean patients with ILD and to evaluate clinical differences between ILD patients with and without OSA.

## Materials and methods

### Study subjects

Eighty-six patients with ILD who underwent respiratory polygraphy (RP) at Haeundae-Paik Hospital, Busan, Republic of Korea, using a portable device (SOMNOcheck micro, level 4, Weinmann Medical Technology, Hamburg, Germany) from December 2017 to April 2019 were included in this study. All patients met diagnostic criteria in international guidelines by the American Thoracic Society (ATS) and European Respiratory Society (ERS) [2, 5]. OSA was defined as an apnea hypopnea index (AHI) of more than 5 events per hour, and the respiratory polygraphy findings were scored following the recommendations in the American Academy of Sleep Medicine (AASM) guideline [13, 14]. This study was approved by the Institutional Review Board of Haeundae-Paik Hospital (approval number: 2019-08-012), and the requirement for written informed consent was waived due to the retrospective nature of this study.

### Clinical information

Clinical and survival data were retrospectively obtained from medical records. Sleep data were scored by an experienced pulmonologist and a registered polysomnographic technologist with 15 years of experience, based on the updated 2012 AASM criteria [14]. Pulmonary function testing, a measurement of the diffusing capacity of the lung for carbon monoxide (DLco), forced expiratory volume in one second, and forced vital capacity (FVC), were performed according to the recommendations of the ATS/ERS [15–17]. The results are expressed as percentages of normal predicted values. The 6-minute walk test was performed according to ATS guidelines [18].

### Respiratory polygraphy

The diagnosis of OSA was based on the results from portable device testing (SOMNOcheck micro, level 4, Weinmann Medical Technology, Hamburg, Germany), which monitors the heart rate, nasal airflow by cannula, and oxygen saturation by pulse oximetry. All recordings were undertaken at the hospital under the control of a sleep technologist. Apnea was defined as a cessation of airflow (≥90% compared to baseline level) for more than 10 seconds, and hypopnea was defined as a clear amplitude reduction of 50–90% in the thermistor of the nasal pressure transducer during sleep that was associated with oxygen desaturation of ≥3% [14, 19]. The arousal index was defined as total number of awakenings per hour of sleep. All traces were scored manually. OSA severity was graded as mild (AHI 5–15 per hour), moderate (AHI 15–30 per hour), and severe (AHI>30 per hour).

### Sleep questionnaires

Prior to the RP test, all patients were asked to complete a sleep questionnaire including the STOP-BANG and Berlin questionnaires. These questionnaires have been widely used as effective and reliable OSA screening tools in clinical practice [20, 21]. In the STOP-BANG questionnaire, the total scores range from 0 to 8, and the risk of OSA can be classified as low (0–2), moderate (3–4), and high (5–8) [20]. The Berlin questionnaire includes scoring parameters for age, sex, weight, height, neck circumference, daytime sleepiness, and body mass index (BMI), and the results classify patients into a high-risk group and a low-risk group for OSA [21].

### Statistical analysis

The data are presented as frequency with percentage for categorical variables and mean ± standard deviation (SD) for continuous variables. The Student’s t-test or Mann-Whitney U test was used to analyze continuous data, and Pearson’s chi-squared test or Fisher’s exact test was used to analyze categorical data. To check for normal distribution of data, we used Shapiro-Wilk’s test. Unadjusted and multivariable analyses, using logistic regression, were performed to identify predictive factors for OSA in patients with ILD. Overall survival was estimated using Kaplan-Meier curves and the log-rank test. For the survival analysis, start date was the date of diagnosis of OSA, and date of vital status ascertainment was 30 April 2019. When performing the survival analysis, we censored the following conditions: (i) survival at a certain time point (1 year) and (ii) follow‐up loss. Risk factors for mortality were analyzed using a Cox proportional hazard model with backward and stepwise elimination. Variables with p < 0.05 in the unadjusted analysis were considered in the multivariable analysis. All statistical analyses were carried out using SPSS 24.0 (IBM Corp, Armonk, USA), and p values less than 0.05 were considered to be statistically significant.

## Results

### Study population

The median follow-up period was 7 months (interquartile range: 4.0–14.3 months). The mean age of the study population was 69.8 years, 64% were men, and 57.9% were ever-smokers. Among ILDs, IPF was the most common (66.3%), followed by connective tissue disease–associated ILD (16.3%), nonspecific interstitial pneumonia (5.8%), and cryptogenic organizing pneumonia (5.8%) (Table 1).

**Table 1:** Comparison of baseline characteristics between OSA and no OSA groups.

Variable	Overall	OSA	No OSA	*P*-value
Patient, no.	86	46	40	
Age, years	69.8 ± 9.8	73.4 ± 6.9	65.7 ± 11.2	<0.001
Male	55 (64.0)	35 (76.1)	20 (50.0)	0.012
Smoking, years	44 (57.9)	28 (66.7)	16 (47.1)	0.085
Weight (kg)	62.9 ± 12.9	65.9 ± 12.6	59.6 ± 12.5	0.023
BMI (kg/m^2^)	24.0 ± 3.6	24.7 ± 3.8	23.2 ± 3.3	0.050
Neck circumference (cm)	36.4 ± 4.0	37.6 ± 3.5	35.1 ± 4.0	0.004
Home oxygen therapy	10 (11.6)	4 (8.7)	6 (15.0)	0.504
CRP (mg/dL)	2.0 ± 4.1	1.1 ± 1.9	3.2 ± 5.5	0.027
Arterial oxygen pressure (mmHg)	82.9 ± 26.7	82.2 ± 26.4	83.8 ± 27.5	0.804
PaCO_2_ (mmHg)	37.6 ± 5.2	39.1 ± 4.9	35.8 ± 4.8	0.003
Pulmonary function				
FVC (%, predicted)	72.7 ± 14.2	74.5 ± 15.3	70.5 ± 12.7	0.192
FEV_1_ (%, predicted)	81.7 ± 16.3	84.9 ± 16.1	77.8 ± 15.8	0.043
DLco (%, predicted)	55.4 ± 15.8	57.5 ± 15.6	52.8 ± 15.8	0.184
Six-minute walk test				
Distance (m)	362.6 ± 96.1	382.6 ± 85.8	338.4 ± 103.5	0.050
Initial SpO_2_ (%)	95.1 ± 3.9	95.3 ± 4.0	95.0 ± 4.0	0.775
Interstitial lung disease				
IPF	57 (66.3)	37 (80.4)	20 (50.0)	0.004
NSIP	5 (5.8)	2 (4.3)	3 (7.5)	
CTD	14 (16.3)	4 (8.7)	10 (25.0)	
COP	5 (5.8)	0 (0.0)	5 (12.5)	
HP	4 (4.7)	3 (6.5)	1 (2.5)	
Underlying disease				
Cardiovascular disease	53 (61.6)	31 (67.4)	22 (55.0)	0.239
Diabetes mellitus	25 (29.1)	19 (41.3)	6 (15.0)	0.007
Chronic kidney disease	5 (5.8)	4 (8.7)	1 (2.5)	0.336
Neurovascular disease	14 (16.3)	9 (19.6)	5 (12.5)	0.376
GERD	12 (14.0)	8 (17.4)	4 (10.0)	0.324
Malignancy	13 (15.1)	6 (13.0)	7 (17.5)	0.565

Data are presented as mean ± standard deviation or number (%), unless otherwise indicated.

ILD, interstitial lung disease; OSA, obstructive sleep apnea; BMI, body mass index; CRP, C-reactive protein; PaCO_2_, partial pressure of carbon dioxide; GAP, gender-age-pulmonary function; FVC, forced vital capacity; FEV_1_, forced expiratory volume in one second; DLco, diffusing capacity of the lungs for carbon monoxide; SpO_2_, saturation of percutaneous oxygen; IPF, Idiopathic pulmonary fibrosis; NSIP, Nonspecific interstitial pneumonia; CTD, Connective tissue disease related; COP, Cryptogenic organizing pneumonia; HP, Hypersensitivity pneumonitis; GERD, gastroesophageal reflux disease

IPF patients were older and had a larger neck circumference than other ILD patients (S1 Table). Among ILD patients, OSA was identified in 46 patients (53.5%). The prevalence of OSA in patients with IPF was higher than in those with other ILDs (64.9% vs. 31.0%, p = 0.03).

### Baseline characteristics

The mean BMI of all patients was 24.0, and the OSA group showed a tendency to have a higher BMI than the non-OSA group (Table 1). The OSA group had a significantly larger neck circumference than the non-OSA group. A history of diabetes mellitus was identified in 29.1% of all patients, and diabetes mellitus was significantly more frequent in the OSA group than the non-OSA group. Lung function (FVC and DLco) did not differ between the OSA group and the non-OSA group.

### Sleep characteristics and questionnaire

Among patients with OSA, mild OSA was the most commonly identified form (52.2%), followed by moderate (34.8%) and severe (13.0%) OSA. The longest apnea in the OSA group was more frequent than in the non-OSA group. The saturation of peripheral oxygen (SpO_2_) and the duration of SpO_2_ less than 90% did not differ between ILD patients with and without OSA. In the STOP-BANG questionnaire, most patients (54.7%) were classified into the moderate risk group for OSA, followed by low risk (29.2%) and high risk (16.3%). In the Berlin questionnaire, 34.9% of participants were in the high risk group for OSA (Table 2) and there was no difference in daytime sleepiness between the OSA and no-OSA groups (OR = 0.48, 95% CI: 0.14–1.63, p = 0.239).

**Table 2:** Comparison of sleep data and questionnaire between OSA and no OSA groups.

Variable	OSA	No OSA	*P*-value
Patient, no.	46	40	
AHI index	16.7 ± 10.2	1.9 ± 1.4	<0.001
Desaturation Index^†^	10.7 ± 11.4	1.4 ± 1.3	<0.001
Mean SpO_2_ (%)	94.3 ± 1.9	94.9 ± 1.6	0.183
Lowest SpO_2_ (%)	80.93 ± 1.9	83.3 ± 6.3	0.338
Duration 90‡ (%)	0.9 ± 2.6	0.8 ± 3.9	0.742
Snoring	3.9 ± 7.4	4.9 ± 8.6	0.724
Longest apnea	37.8 ± 13.8	18.1 ± 12.7	<0.001
Arousal index	11.5 ± 9.8	8.6 ± 5.6	0.402
SBQ risk			
Low risk	11 (23.9)	14 (35.0)	0.528
Moderate risk	27 (58.7)	20 (50.0)	
High risk	8 (17.4)	6 (15.0)	
BQ risk	17(37.0)	13 (32.5)	0.665

Data are presented as mean ± standard deviation or number (%), unless otherwise indicated.

ILD, interstitial lung disease; OSA, obstructive sleep apnea; AHI, apnea-hypopnea index; SpO_2_, saturation of percutaneous oxygen; SBQ, STOP-BANG questionnaire; BQ, Berlin questionnaire

^†^Desaturation index–the average number of desaturation episodes per hour of sleep

‡Duration90 –duration less than 90% SpO_2_ of total sleep

In the unadjusted logistic regression analyses, the Berlin questionnaire was not a significant risk factor for OSA in patients with ILD, and moderate and high SBQ risk were not associated with OSA occurrence compared with low SBQ risk (Table 3).

**Table 3:** Predictive factors for OSA in patients with ILD assessed using a logistic regression model.

Variable	Unadjusted analysis		Multivariable analysis	
	OR (95% CI)	*P-*value	OR (95% CI)	*P-*value
Age	1.11 (1.04–1.18)	0.001	1.12 (1.04–1.20)	0.002
Male	3.18 (1.27–7.97)	0.013		
Smoking	2.25 (0.89–5.70)	0.087		
Weight	1.04 (1.00–1.08)	0.027	1.05 (1.01–1.10)	0.024
BMI	1.13 (1.00–1.29)	0.056		
Neck circumference	1.19 (1.05–1.35)	0.006		
CRP	0.83 (0.69–0.99)	0.038		
Arterial oxygen pressure	1.00 (0.98–1.02)	0.801		
Pulmonary function				
FVC	1.02 (0.99–1.05)	0.193		
FEV_1_	1.03 (1.00–1.06)	0.048		
DLco	1.02 (0.99–1.05)	0.184		
Six-minute walk test				
Distance	1.01 (1.00–1.01)	0.054		
Initial SpO_2_	1.02 (0.91–1.15)	0.740		
Lowest SpO_2_	1.02 (0.97–1.07)	0.458		
Interstitial lung disease				
IPF	4.11 (1.58–10.70)	0.004		
NSIP	0.56 (0.09–3.54)	0.538		
CTD	0.29 (0.08–1.00)	0.049		
COP	0.00 (0.00–0.00)	0.999		
HP	2.72 (0.27–27.26)	0.395		
Underlying disease				
Cardiovascular disease	1.69 (0.70–4.06)	0.240		
Diabetes mellitus	3.99 (1.40–11.37)	0.010	3.94 (1.20–12.99)	0.024
Chronic kidney disease	3.71 (0.40–34.69)	0.250		
Neurovascular disease	1.70 (0.52–5.58)	0.379		
GERD	1.89 (0.52–6.84)	0.329		
Malignancy	0.71 (0.22–2.31)	0.566		
SBQ risk				
Low risk	Ref			
Moderate risk	1.72 (0.65–4.57)	0.278		
High risk	1.70 (0.45–6.36)	0.433		
BQ risk	1.22 (0.50–2.97)	0.666		

OSA, obstructive sleep apnea; ILD, interstitial lung disease; BMI, body mass index; CRP, C-reactive protein; GAP, gender-age-pulmonary function; FVC, forced vital capacity; FEV_1_, forced expiratory volume in one second; DLco, diffusing capacity of the lungs for carbon monoxide; SpO_2_, saturation of percutaneous oxygen; IPF, Idiopathic pulmonary fibrosis; NSIP, Nonspecific interstitial pneumonia; CTD, Connective tissue disease related; COP, Cryptogenic organizing pneumonia; HP, Hypersensitivity pneumonitis; GERD, gastroesophageal reflux disease; SBQ, stop bang questionnaire; BQ, berlin questionnaire

### Predictive factors for OSA

In the unadjusted logistic regression analysis, older age was a significant risk factor for OSA in patients with ILD, along with male sex, higher body weight, larger neck circumference, low level of C-reactive protein, diagnosis of IPF, and history of diabetes mellitus (Table 3). In the multivariable analysis, older age (OR 1.11, 95% CI: 1.04–1.10, p = 0.002), higher body weight (OR 1.05, 95% CI: 1.01–1.10, p = 0.012), and diabetes mellitus (OR 4.03, 95% CI: 1.26–12.91, p = 0.019) were independent risk factors for OSA.

### Survival analysis

**Fig 1:**
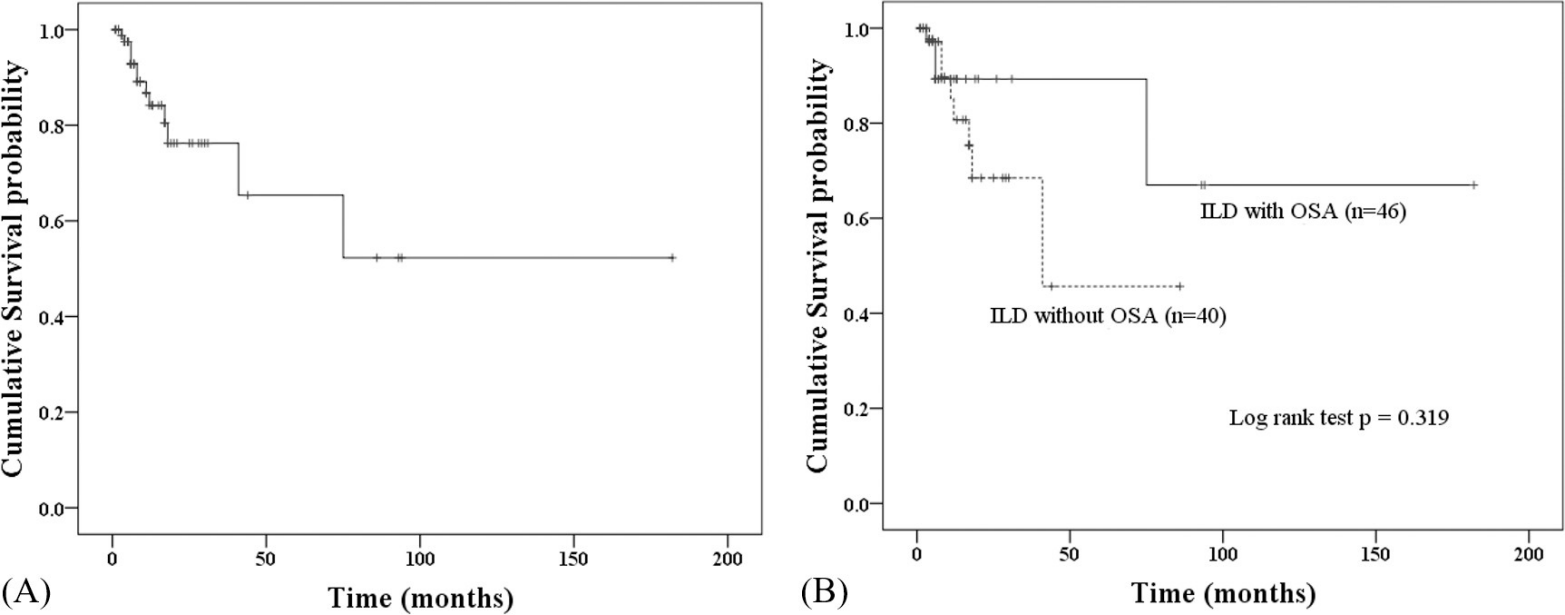
Kaplan–Meier survival curves in patients with interstitial lung disease. (A) Survival curves of total patients with interstitial lung disease (ILD); (B) Comparison of survival curves between ILD patients with and without obstructive sleep apnea.

During the follow-up period, thirteen patients (15.1%) died, and 38.5% of them had OSA. The most common cause of death was acute respiratory failure caused by an acute exacerbation of ILD and pneumonia (69.2%). The one-year survival rate of all patients was 84.1% (Fig 1A). The one-year survival rate did not differ between patients with and without OSA (89.3% vs. 80.7%, p = 0.319) (Fig 1B).

In the multivariable Cox analysis, IPF was the only a significant prognostic factor for one-year mortality (HR 7.92, 95% CI: 1.01–61.83, p = 0.048) in patients with ILD; however, OSA was not (Table 4).

**Table 4:** Prognostic factor for mortality in patients with ILD assessed using Cox proportional hazards model.

Variable	Unadjusted analysis			Multivariable analysis		
	HR	95%CI	*P-*value	HR	95%CI	*P-*value
Age	1.05	(0.97–1.12)	.235			
Male	1.51	(0.49–4.69)	.476			
Smoking	1.25	(0.37–4.26)	.722			
Weight	1.01	(0.97–1.05)	.769			
BMI	1.03	(0.91–1.18)	.625			
Neck circumference	1.05	(0.91–1.21)	.504			
CRP	0.91	(0.72–1.14)	.418			
Arterial oxygen pressure	1.01	(0.99–1.03)	.307			
Pulmonary function						
FVC	0.96	(0.93–1.00)	.080			
FEV_1_	0.99	(0.95–1.02)	.535			
DLco	0.97	(0.94–1.01)	.118			
Six-minute walk test						
Distance	1.00	(0.99–1.00)	.160			
Initial SpO_2_	0.91	(0.80–1.03)	.139			
Lowest SpO_2_	1.01	(0.96–1.06)	.749			
Interstitial lung disease						
IPF	7.92	(1.01–61.83)	.048	7.92	(1.01–61.83)	.048
CTD	0.02	(0.00–4.39)	.161			
COP	5.56	(0.63–49.22)	.123			
Underlying disease						
Cardiovascular disease	0.91	(0.29–2.79)	.866			
Diabetes mellitus	2.66	(0.81–8.78)	.108			
Neurovascular disease	3.21	(0.94–11.00)	.064			
GERD	1.44	(0.39–5.27)	.586			
Malignancy	1.66	(0.45–6.13)	.450			
SBQ risk						
Low risk		Ref				
Moderate risk	1.03	(0.30–3.57)	.965			
High risk	0.58	(0.06–5.22)	.625			
BQ risk	0.64	(0.19–2.09)	.457			
OSA	0.56	(0.18–1.77)	.327			

ILD, interstitial lung disease; BMI, body mass index; CRP, C-reactive protein; FVC, forced vital capacity; FEV_1_, forced expiratory volume in one second; DLco, diffusing capacity of the lungs for carbon monoxide; SpO_2_, saturation of percutaneous oxygen; IPF, Idiopathic pulmonary fibrosis; NSIP, Nonspecific interstitial pneumonia; CTD, Connective tissue disease related; COP, Cryptogenic organizing pneumonia; GERD, gastroesophageal reflux disease; SBQ, stop bang questionnaire; BQ, berlin questionnaire; OSA, obstructive sleep apnea

## Discussion

In our study, the prevalence of OSA in Korean patients with ILD was 53.5%, and IPF patients showed a higher prevalence of OSA than other ILD patients. Older age, higher body weight, and diabetes mellitus were independent factors predicting OSA. However, one-year survival did not differ between ILD patients with and without OSA.

In our study, a half of Korean patients with ILD had OSA, and OSA was more common in IPF patients. Previous studies support our results. In 50 patients with ILD (17 IPF, 15 sarcoidosis, and 18 scleroderma related ILD), Pihtili et al. found an OSA prevalence of 68% in all patients, and OSA was most common in IPF patients (82.3%), followed by sarcoidosis (66.6%), and scleroderma (55.5%) patients [22].

Despite increasing recognition of the high prevalence of OSA in patients with ILD, the mechanisms of association between ILD and OSA have not yet been clarified. One hypothesis suggests that ILD might aggravate the occurrence of OSA [23, 24]. The lower lung volumes observed in ILD might promote OSA by reducing traction in the trachea and increasing pharyngeal collapsibility. Other researchers have hypothesized that OSA might cause early alveolar epithelial cell injury during sleep through cyclic hypoxia and re-oxygenation [25, 26]. In a previous study of 11 OSA patients and 10 controls using Krebs von den Lungen 6 (KL-6), a biomarker for the diagnosis and prognosis of ILD, the KL-6 level was significantly higher in the OSA group than the controls (317 U/mL vs. 226 U/mL, p = 0.03). Those authors suggested that circulating KL-6 levels are elevated in OSA patients, possibly reflecting increased alveolar wall permeability and alveolar injury [27].

In our study, older age, higher weight, and diabetes mellitus were significant predictive factors for OSA in patients with ILD. Previous results also support those findings [28, 29]. In 50 patients with IPF, Lancaster et al. reported that BMI correlated positively with OSA (*r* = 0.30; p = 0.05) [28]. In 49 patients with ILD, none of whom were obese (excluding BMI over 30 kg/m^2^), Pereira et al. also reported that excess weight might predict a higher risk of OSA in patients with ILD [29]. In our study, diabetes mellitus was a strong risk factor for OSA. Reutrakul et al. suggested that the relationship between OSA and diabetes mellitus could be bidirectional because diabetic neuropathy can affect upper airway neural reflexes and promote sleep-disordered breathing. In the other direction, laboratory-based experiments in healthy human subjects have demonstrated that sleep restriction, sleep fragmentation, and intermittent hypoxemia can lead to the dysregulation of glucose metabolism [30].

Several sleep questionnaires have been used as screening tools for OSA [31]. Among them, the STOP-BANG and Berlin questionnaires have been widely used for screening OSA in patients with ILD. In 77 patients with ILD, Xiao et al. reported that a STOP-BANG score of more than 3 points (moderate to high score) could help to predict OSA (OR = 6.29, 95% CI: 2.21–17.9, p < 0.01) [32]. Among 49 patients with ILD, Mavroudi et al. reported that the Berlin questionnaire correlated positively with OSA (Spearman correlation: 0.484, p = 0.036) [33]. However, the role of sleep questionnaires in patients with ILD is not yet well defined. In our study, the STOP-BANG and Berlin questionnaires did not effectively predict OSA in patients with ILD. In the near future, large-scale studies are needed to develop more effective screening tools for OSA in patients with ILD.

In recent studies, OSA and hypoxemia have been shown to correlate with disease-related mortality in ILD patients [34]. In a prospective, single-cohort study of 31 IPF patients, Kolilekas et al. reported that the lowest category of SpO_2_ correlated directly with survival (HR 0.897, 95% CI: 0.827–0.972, p = 0.009), but AHI did not. Those authors suggested that the association between apnea events and lung damage resulted from severe intermittent oxygen desaturation during sleep, which aggravated pulmonary artery hypertension and influenced the survival of IPF patients [35]. In our study, OSA was not a significant predictor of one-year mortality. We also found no differences in desaturation, including lowest recorded SpO_2_ and duration of SpO_2_ less than 90% for 5 minutes during sleep, between ILD patients with and without OSA. Therefore, we suggest that hypoxemia, rather than OSA itself, might play an important role in survival. In our study, a diagnosis of IPF was the only independent predictive factor for one-year mortality, regardless of OSA.

This study has some limitations. First, it was a retrospective study conducted in a single center with a relatively small number of patients. However, the baseline characteristics of our subjects were similar to those of patients in previous reports [29, 33, 36, 37]. Second, RP was performed using a level 4 device instead of a full level 1, gold standard polysomnography (PSG) test. Therefore, we could not obtain more detailed data about sleep such as an electroencephalogram, electrooculogram, and electromyogram. Nonetheless, our level 4 device was well validated for diagnosing OSA in previous studies [38]. Third, the follow-up period was too short, and we had difficulty evaluating the predictive capacity of OSA for one-year mortality in patients with ILD. A long-term follow-up study might be useful to evaluate the role of OSA as a prognostic factor in patients with ILD.

## Conclusion

Half of Korean patients with ILD had OSA, and OSA was more common in IPF patients than in those with other ILDs. Older age, higher body weight, and diabetes mellitus indicate a high risk for OSA, and a diagnosis of IPF, but not OSA itself, was an independent risk factor for one-year mortality in ILD patients.

## Supporting information

S1 Table Comparison of baseline characteristics between patients with patients with IPF and other ILD. (DOCX)
