# Supplementary material for: Obstructive sleep apnea in patients with interstitial lung disease: Prevalence and predictive factors
Source: PLoS One. 2020 Oct 5;15(10):e0239963. doi: 10.1371/journal.pone.0239963 (PMC7535061; doi:10.1371/journal.pone.0239963)
Supplement: S1 Table — (DOCX) [file pone.0239963.s001.docx]

| **S1 Table. Comparison of baseline characteristics between patients with patients with IPF and other ILD.** | | | |
| --- | --- | --- | --- |
| **Variable** | **Group** | |  |
|  | **IPF** | **Other ILD** | ***P*-value** |
|  | **(n = 57)** | **(n = 29)** |  |
| **Age, years** | 72.5 ± 7.2 | 64.8 ± 12.2 | 0.004 |
| **Male** | 47 (82.5) | 8 (27.6) | <0.001 |
| **Smoking** | 38 (74.5) | 6 (24.0) | <0.001 |
| **Weight (kg)** | 65.2 ± 12.8 | 58.6 ± 12.1 | 0.024 |
| **BMI (kg/m^2^)** | 24.1 ± 3.6 | 23.7 ± 3.8 | 0.587 |
| **Neck circumference (cm)** | 37.6 ± 3.6 | 34.2 ± 3.7 | <0.001 |
| **C-reactive protein (mg/dL)** | 3.9 ± 0.5 | 4.3 ± 0.8 | 0.010 |
| **Arterial oxygen pressure (mmHg)** | 85.7 ± 26.9 | 75.99 ± 25.6 | 0.162 |
| **Pulmonary function** |  |  |  |
| FVC (%, predicted) | 72.4 ± 14.8 | 73.3 ± 13.4 | 0.798 |
| FEV1 (%, predicted) | 82.7 ± 16.7 | 79.54 ± 15.3 | 0.404 |
| DLco (%, predicted) | 55.4 ± 17.1 | 55.4 ± 12.9 | 0.980 |
| **Six-minute walk test** |  |  |  |
| Distance (m) | 361.7 ± 96.9 | 364.9 ± 96.4 | 0.899 |
| Initial SpO2 (%) | 95.3 ± 3.4 | 94.67 ± 5.1 | 0.941 |
| Lowest SpO2 (%) | 89.3 ± 7.5 | 85.3 ± 12.4 | 0.220 |
| **Polysomnographic data** |  |  |  |
| Obstructive sleep apnea | 37 (64.9) | 9 (31.0) | 0.003 |
| AHI index | 12.4 ± 11.3 | 5.1 ± 6.7 | <0.001 |
| Mean SpO2 (%) | 94.6 ± 1.9 | 94.6 ± 1.5 | 0.979 |
| Lowest SpO2 (%) | 81.7 ± 8.6 | 82.7 ± 6.3 | 0.812 |
| Duration <90% for 5 min(min) | 1.2 ± 3.8 | 0.5 ± 1.9 | 0.215 |
| Snoring (%) | 3.9 ± 6.8 | 5.2 ± 9.9 | 0.427 |
| Longest apnea(second) | 32.8 ± 14.7 | 21.14 ± 17.3 | 0.002 |
| Arousal index | 10.2 ± 8.9 | 9.9 ± 6.6 | 0.642 |
| **SBQ risk** |  |  |  |
| Low risk | 12 (21.1) | 13 (44.8) | 0.093 |
| Moderate risk | 35 (61.4) | 12 (41.4) |  |
| High risk | 10 (17.5) | 4 (13.8) |  |
| **BQ risk** | 21 (36.8) | 9 (31.0) | 0.593 |

Data are presented as mean ± standard deviation or number (%), unless otherwise indicated.

IPF, idiopathic pulmonary fibrosis; ILD, interstitial lung disease; BMI, body mass index; FVC, forced vital capacity; FEV1, forced expiratory volume in one second; DLco, diffusing capacity of the lungs for carbon monoxide; SpO_2,_ saturation of percutaneous oxygen; SBQ, stop bang questionnaire; BQ, berlin questionnaire
